# Antibacterial and Antibiofilm Activity of AMP-15 Against *Staphylococcus aureus* and Its Potential Application in Food Matrices

**DOI:** 10.3390/foods15213990

**Published:** 2026-11-06

**Authors:** Elifeiye Aini, Qiong Jia, Ning Wang, Wei Huang, Lijuan Zhang, Jing Zhu, Rubing Du, Qing Xu, Yonghong Fan, Zhidong Zhang

**Affiliations:** 1 College of Life Science and Technology, Xinjiang University, Urumqi 830000, China; 107552401044@stu.xju.edu.cn; 2 Xinjiang Key Laboratory of Special Environmental Microbiology, National Collection of Microbial Resource for Fertilizer (Xinjiang), Institute of Microbiology, Xinjiang Academy of Agricultural Sciences, Urumqi 830000, China; jiaqiong7270@163.com (Q.J.); wangning950203@163.com (N.W.); ryan_huangwei1991@126.com (W.H.); zhanglijuan@xaas.ac.cn (L.Z.); zhujing2020@hotmail.com (J.Z.); 3 State Key Laboratory of Microbial Technology, School of Food Science and Pharmaceutical Engineering, Nanjing Normal University, Nanjing 210000, China; rubingdu@nnu.edu.cn (R.D.); xu_qing@njnu.edu.cn (Q.X.); 4 College of Food Sciences and Pharmacy, Xinjiang Agricultural University, Urumqi 830000, China

**Keywords:** cationic peptide, membrane disruption, foodborne pathogen, antibacterial mechanism, food preservation

## Abstract

*S. aureus* is a leading foodborne pathogen, and its biofilms limit the effectiveness of conventional contamination control, creating a need for new preservatives. This study evaluated the novel short cationic antimicrobial peptide AMP-15 (18 amino acids, +6 net charge, amphipathic α-helix) against planktonic and biofilm *S. aureus*. Bactericidal kinetics and mechanism were examined by viability assays, SYTO9/PI staining, scanning and transmission electron microscopy, ROS measurement (DCFH-DA), and molecular docking against *S. aureus* thymidylate kinase (TMK). Antibiofilm activity was quantified as inhibition of biofilm formation and reduction in mature biofilm biomass and viable cells, with hemolysis, cytotoxicity, pH and temperature stability, and chicken and milk food models used to assess safety and applicability. AMP-15 produced rapid, concentration-dependent killing (MIC = 12.5 μg/mL) and reduced viable counts by approximately 2.0–2.5 log_10_ CFU/mL within 2 h at 1×–2× MIC, accompanied by membrane disruption, surface collapse, intracellular disorganization, and elevated ROS, and was predicted to bind thymidylate kinase with a binding affinity of −6.4 kcal/mol. It inhibited biofilm formation by up to ~80% at 2× MIC and achieved 40–60% eradication of mature biofilm biomass, caused low hemolysis at ≤50 μg/mL but some cytotoxicity at high concentrations (IC_50_ = 58.55 μg/mL against 293T cells), remained active across the tested pH and temperature ranges, and significantly reduced *S. aureus* by ~1.4–1.5 log_10_ CFU/g in chicken and ~1.2 log_10_ CFU/mL in sterilized milk. AMP-15 therefore shows strong anti-staphylococcal and antibiofilm potential as a food preservative, although safety and target validation require further study.

## 1. Introduction

*Staphylococcus aureus* is one of the most common and hazardous foodborne pathogens in food systems. It can contaminate a wide range of foods, including meat products, dairy products, ready-to-eat foods, and fruit and vegetable products [1,2,3]. This bacterium can survive over a broad range of temperature, pH, and salt concentrations, showing strong environmental adaptability during food processing and storage. In addition, it can produce heat-stable enterotoxins, thereby increasing food safety risks. At present, strategies used in the food industry to control *S. aureus* contamination mainly include heat treatment, chemical preservatives, and chlorine-containing disinfectants [4,5]. Although these methods contribute to food safety to some extent, they also have clear limitations. For example, heat treatment may affect the sensory quality and nutritional properties of foods; the use of chemical preservatives is increasingly challenged by consumer demand for “clean-label” products and natural ingredients [6]; and the actual efficacy of conventional disinfectants in complex processing environments may be limited by organic residues, surface materials, and the attachment state of microorganisms. Therefore, the development of safe, effective, and food-compatible natural antibacterial agents remains an important requirement for food preservation and microbial control in food-processing environments products and natural ingredients.

In addition to planktonic cells, the persistent contamination of *S. aureus* in food-processing environments is closely associated with its ability to form biofilms [7]. *S. aureus* can adhere to food-contact surfaces such as stainless steel, plastics, and rubber, and form structured biofilms by secreting extracellular polymeric substances [8], including polysaccharides, proteins, and extracellular DNA [9]. Bacteria embedded in biofilms generally exhibit stronger environmental tolerance than planktonic cells and can resist desiccation, acid and alkaline stress, heat treatment, and disinfectants, thereby reducing the effectiveness of routine cleaning and sanitization procedures [10]. The presence of biofilms may also lead to persistent contamination on processing equipment surfaces and increase the risk of cross-contamination between different batches of food products [9,11]. Therefore, evaluating only the bactericidal effect of an antibacterial agent against planktonic cells is insufficient to reflect its potential application value in food-processing scenarios. An ideal food-preservation candidate should not only rapidly inhibit or kill target bacteria but also interfere with biofilm formation or weaken mature biofilms.

In recent years, with increasing consumer interest in natural preservatives and clean-label foods, natural or bio-derived antibacterial strategies, including plant-derived bioactive compounds, bacteriophages, enzyme preparations, and antimicrobial peptides, have attracted considerable attention. Among them, antimicrobial peptides (AMPs) are typically characterized by short chain length, cationic charge, and amphipathicity. They can exert antibacterial effects by interacting with bacterial cell membranes, disrupting membrane integrity, or further affecting intracellular physiological processes [12,13]. Compared with some conventional antibacterial agents, AMPs generally have rapid bactericidal activity, diverse modes of action, and a lower likelihood of rapidly inducing resistance through single-target mutations. Therefore, they are regarded as promising candidate molecules for the development of novel food preservatives [14,15,16]. The bacteriocin nisin has already been successfully commercialized as a food preservative, demonstrating that peptide-based antibacterial agents can be practically applied in food systems [17]. However, research on many novel AMPs remains largely limited to in vitro antibacterial activity assays under culture-medium conditions. Systematic investigations of their efficacy, safety, stability, and antibiofilm activity in complex food matrices are still relatively insufficient [18,19]. Components in foods, such as proteins, lipids, salt ions, and endogenous enzymes, may weaken the activity of AMPs. Therefore, it is necessary to further verify their application potential in real or semi-realistic food models [20,21,22,23].

In the present study, using food-related *S. aureus*, we systematically evaluated the antibacterial activity, mechanism of action, antibiofilm capacity, biocompatibility and stability of the antimicrobial peptide AMP-15, and further validated its inhibitory efficacy in chicken and milk contamination models, to provide experimental evidence for its development as a novel peptide-based food preservative.

## 2. Materials and Methods

### 2.1. Materials and Bacterial Strains

Prodigal with optimized parameters was used to predict short open reading frames (smORFs) ranging from 10 to 100 amino acids (aa). A total of 21 physicochemical and structural features were extracted from each peptide sequence, and an imbalanced dataset with a positive-to-negative sample ratio of approximately 1:50 was constructed to train a random forest classifier for antimicrobial peptide identification. The classifier achieved both specificity and precision of 0.998 on the independent test set. Candidate sequences obtained from the initial screening were subjected to de-redundancy, sequence clustering and homology comparison. Sequences matching known antimicrobial peptides were removed by searching public databases such as DRAMP, and candidates with high toxicity were further eliminated using a hemolytic activity prediction model. Finally, a novel cationic antimicrobial peptide AMP-15 with the amino acid sequence FNIIRKFVLLKIIRRFLR was obtained. AMP-15 was synthesized and purified via solid-phase peptide synthesis by Nanjing Peptide Valley Biotechnology Co., Ltd. (Nanjing, China), with a purity ≥ 95%. Prior to experiments, AMP-15 was dissolved in sterile PBS (pH 7.4) to prepare a stock solution of 1 mg/mL, which was stored at 4 °C for short-term use and diluted to the required working concentrations before each assay.

The tested bacterial strains included *Staphylococcus aureus* CMCC(B)186335, *Klebsiella pneumoniae* BNCC102997, *Bacillus cereus* ATCC 11778 and *Escherichia coli* CMCC(B)44102, which were provided by our laboratory. Each strain was activated by streaking on Mueller–Hinton agar plates. A single colony was then inoculated into LB broth and cultured at 37 °C with shaking at 180 rpm until the logarithmic growth phase (about 6 h). For quantitative assays, the bacterial suspension was generally adjusted to an OD_600_ of 0.6–0.8.

### 2.2. Physicochemical Property and Structural Analysis of AMP-15

The physicochemical properties of AMP-15 were calculated using the HeliQuest online tool, including molecular weight, net charge, mean hydrophobicity, hydrophobic moment, and grand average of hydropathicity (GRAVY). A helical wheel projection was also generated. The three-dimensional structure of AMP-15 was visualized using PyMOL 3.0.3.

### 2.3. Determination of Antibacterial Activity

#### 2.3.1. Inhibition Zone Assay

The bacterial suspension was diluted to approximately 10^6^ CFU/mL, and 100 μL of the suspension was evenly spread onto the surface of Mueller–Hinton agar plates. Sterile filter paper disks with a diameter of 5 mm were placed on the agar surface, and 20 μL of AMP-15 solution (4 mg/mL) was added to each disk. PBS was used as the negative control, and ampicillin (100 μg/mL) sodium was used as the positive control. After incubation at 37 °C for 16 h–18 h, the diameter of the inhibition zone was measured.

#### 2.3.2. Minimum Inhibitory Concentration (MIC) and Growth Curve Analysis

The MIC was determined by the broth microdilution method following the Clinical and Laboratory Standards Institute (CLSI) guidelines, using Mueller–Hinton broth (MHB) and Luria–Bertani (LB) broth for MIC measurement. Briefly, AMP-15 was serially two-fold diluted and mixed with the *S. aureus* bacterial suspension in 96-well plates, followed by incubation at 37 °C for 18–24 h. The bacterial suspension without AMP-15 was used as the growth control, and sterile medium was used as the blank control. After incubation, bacterial growth was evaluated by visual observation of turbidity and measurement of OD_450_. The MIC was defined as the lowest concentration of AMP-15 at which no visible bacterial growth was observed and the OD_450_ value was close to that of the blank control.

#### 2.3.3. Time–Kill Curve Assay

The bacterial suspension, at approximately 10^6^ CFU/mL, was mixed with AMP-15 at final concentrations of 1/4×, 1/2×, 1×, and 2× MIC. PBS and ampicillin (100 μg/mL) were used as the negative and positive controls, respectively. Samples were collected at 0, 1, 2, 3, 5, 7, and 9 h, serially diluted, and spread onto agar plates, followed by incubation at 37 °C for colony counting.

### 2.4. Analysis of Antibacterial Mechanisms

#### 2.4.1. Cell Membrane Integrity Assay

*S. aureus* cells, at approximately 10^6^ CFU/mL, were treated with AMP-15 at 1/2× MIC and 1× MIC. After treatment, PI (20 μΜ)/SYTO 9 (5 μM) staining solution was added, and the samples were incubated in the dark for 15 min. Acquire images immediately after incubation and complete shooting within 30 min. Untreated bacterial cells were used as the control group. Fluorescence changes were observed using a fluorescence microscope. Micrographs were exported, and the fluorescence intensity was quantitatively analyzed using ImageJ v1.54g.

#### 2.4.2. Scanning Electron Microscopy (SEM)

Log-phase *S. aureus* cells were treated with AMP-15 at 1/2× and 1× MIC for 6 h, with untreated cells serving as the control. Bacterial pellets were collected, washed with 0.01 M PBS (pH 7.4), and fixed with 1% OsO_4_ in PBS for 2 h. Samples were then dehydrated through a graded ethanol series (30–100%), critical-point-dried, sputter-coated with gold, and observed under SEM for surface morphology changes.

#### 2.4.3. Transmission Electron Microscopy (TEM)

Log-phase *S. aureus* cells were treated with AMP-15 at 1/2× and 1× MIC for 6 h, with untreated cells serving as the control. Bacterial pellets were fixed with electron microscopy fixative at 4 °C for 3 h, washed with PBS, embedded in 1% agarose, and post-fixed with 1% OsO_4_ for 2 h. Samples were then dehydrated through graded ethanol, acetone-transitioned, infiltrated with EMBed 812 resin, and polymerized at 65 °C for 2 days. Ultrathin sections (60–80 nm) were stained with uranyl acetate and lead citrate for TEM observation.

#### 2.4.4. Reactive Oxygen Species (ROS) Assay

Log-phase *S. aureus* cells were treated with AMP-15 (1/4×–2× MIC; untreated control) at 30 °C for 2 h. Cells were collected, washed, resuspended in PBS containing 10 μM DCFH-DA, and incubated at 30 °C for 20 min in the dark (with gentle mixing every 3–5 min). After three PBS washes, fluorescence intensity was measured (Ex/Em 488/525 nm), and images were captured by microscopy to assess intracellular ROS levels. Fluorescence intensity was performed using ImageJ software, as described in Section 2.4.4.

#### 2.4.5. Molecular Docking

TMK catalyzes the phosphorylation of dTMP to dTDP, is a key enzyme in bacterial DNA synthesis, and has been validated as an antibacterial target. The protein sequence of *S. aureus* thymidylate kinase (TMK) was obtained from UniProt, and its 3D structure from RCSB PDB. The structure was preprocessed in Discovery Studio 2019 (removing water, adding hydrogens, completing missing atoms, charge correction, optimization) and saved as PDB. AMP-15 3D structure was built and energy-minimized in Discovery Studio 2019. Using AutoDock Tools 1.5.6, receptor and ligand were prepared (polar hydrogens, Gasteiger charges, rotatable bonds) and converted to PDBQT. Docking was performed with AutoDock Vina 1.2.6. The binding mode with low binding energy and reasonable conformation was selected. Interactions and residues were visualized with PyMOL 3.1 and Discovery Studio 2019.

### 2.5. Biofilm Assays

#### 2.5.1. Inhibition of Biofilm Formation

The *S. aureus* bacterial suspension (5 μL, OD_450_ = 0.4) was inoculated into sterile 96-well plates, and 195 μL of AMP-15 was added simultaneously to final concentrations of 0 (without AMP), 1/4× MIC, 1/2× MIC, 1× MIC, and 2× MIC. The plates were statically incubated at 37 °C for 0, 10, 24, and 48 h. After incubation, the supernatant was gently discarded, and the wells were slowly washed three times with PBS to remove non-adherent planktonic cells. Methanol was then added for fixation for 30 min, after which the fixative was discarded and the plates were air-dried. Crystal violet staining solution was added to each well for 10–15 min. Excess stain was removed, and the wells were air-dried again. Ethanol or glacial acetic acid was then added to dissolve the dye bound to the biofilm. The absorbance was measured at 595 nm (OD_595_), and the biofilm biomass and inhibition rate were predicted accordingly.

#### 2.5.2. Eradication of Mature Biofilms

*S. aureus* was statically cultured in 96-well plates at 37 °C for 24 h to allow mature biofilm formation. The supernatant was discarded, and the wells were gently washed with PBS to remove non-adherent bacteria. AMP-15 was then added at final concentrations of 0, 1/4× MIC, 1/2× MIC, 1× MIC, and 2× MIC, and the biofilms were treated for 0, 6, 12, and 24 h. After treatment, part of the samples was analyzed using the crystal violet staining method to determine residual biofilm biomass. For the remaining samples, the biofilms were scraped or mildly ultrasonically dispersed. The absorbance was measured at 595 nm (OD595), followed by serial dilution and plate counting to evaluate changes in the number of viable cells within mature biofilms.

### 2.6. Evaluation of Cytocompatibility and Stability

#### 2.6.1. Hemolytic Activity Assay

A 2% mouse erythrocyte (purchased from Shanghai Yuanye Bio-Technology Co., Ltd., Shanghai, China) suspension was centrifuged at 1500 rpm for 5 min. The supernatant was discarded, and the erythrocytes were washed with PBS and resuspended. This suspension was mixed with AMP-15 (final concentrations 3.125–400 μg/mL). PBS was used as negative control and 1% Triton X-100 as positive control. After incubation at 37 °C for 1 h, samples were centrifuged again (1500 rpm, 5 min), and the supernatant was measured for absorbance at the corresponding wavelength. The hemolysis rate was calculated using the following equation: Hemolysis (%) = (A_sample − A_negative control)/(A_positive control − A_negative control) × 100%.

#### 2.6.2. Cytotoxicity Assay

293T cells (mammalian cells) were seeded in 96-well plates at 5 × 10^4^ cells/mL in complete medium and incubated overnight (37 °C, 5% CO_2_). After removing non-adherent cells, fresh medium with AMP-15 (0, 3.125, 6.25, 12.5, 25, 50, 100, 200, 400 μg/mL) was added. Following 12 h treatment, 10 μL CCK-8 reagent was added, incubated for 2 h in the dark, and absorbance measured at 450 nm. Cell viability was calculated as experimental/control absorbance, and IC_50_ was fitted.

#### 2.6.3. Stability Analysis

The pH stability of AMP-15 was evaluated using a series of buffers with different pH values, glycine–HCl buffer (pH 2), citric acid–sodium citrate (pH 4), sodium phosphate buffer (pH 6), PBS (pH 7) Tris-HCl buffer (pH 8), glycine–NaOH buffer (pH 10) and disodium hydrogen phosphate–sodium hydroxide buffer (pH 12), incubated at room temperature for 2 h, neutralized, and serially diluted. MIC against *S. aureus* was re-determined by broth microdilution (untreated AMP-15 as control). For thermal stability, AMP-15 was incubated at 4, 40, 60, 80, and 100 °C for 30 min, returned to room temperature, serially diluted, and MIC determined similarly (untreated control). All experiments were repeated at least three times.

### 2.7. Chicken Contamination Model

Fresh chicken breast was purchased from a local supermarket and cut into ~1 cm^3^ pieces, sterilized by sequential immersion in 0.5% sodium hypochlorite and 75% ethanol, rinsed with PBS, and exposed to UV for 1.2 h. A bacterial suspension (5 × 10^3^ CFU/mL) was evenly spread onto the chicken surface, followed by incubation at 25 °C for 2 h to establish contamination. AMP-15 (2× MIC) or PBS (control) was applied, and samples were incubated at 25 °C for another 32 h. After treatment, chicken pieces were weighed, homogenized in PBS, serially diluted, spread on LB agar, and incubated at 37 °C for colony counting. Results were normalized to weight and expressed as log_10_(CFU/g).

### 2.8. Sterilized Milk Contamination Model

Commercially packaged fresh milk was purchased from a local supermarket; 30 mL was dispensed into 50 mL conical flasks, sealed with sterile sealing film, autoclaved at 105 °C for 15 min, cooled, and stored at 4 °C until use. A bacterial suspension of 5 × 10^3^ CFU/mL (100 μL) was inoculated into the sterilized milk and incubated at 25 °C for 2 h. AMP-15 was then added at 2× MIC, with PBS used as the control, and the samples were further incubated at 25 °C for 32 h. After treatment, samples were collected, serially diluted, and spread onto LB agar plates, followed by incubation at 37 °C for colony counting. The results were expressed as log_10_(CFU/mL).

### 2.9. Statistical Analysis

All experiments were independently repeated at least three times, and data are presented as the mean ± standard deviation. Statistical analysis was performed using GraphPad Prism 10.1.2. Comparisons between two groups were conducted using Student’s *t*-test, while comparisons among multiple groups were performed using two-way analysis of variance (two-way ANOVA). A value of * *p* < 0.05, ** *p* < 0.01, *** *p* < 0.001, or **** *p* < 0.0001 was considered statistically significant.

## 3. Results

### 3.1. Physicochemical Properties and Structural Characteristics of AMP-15

**Figure 1:**
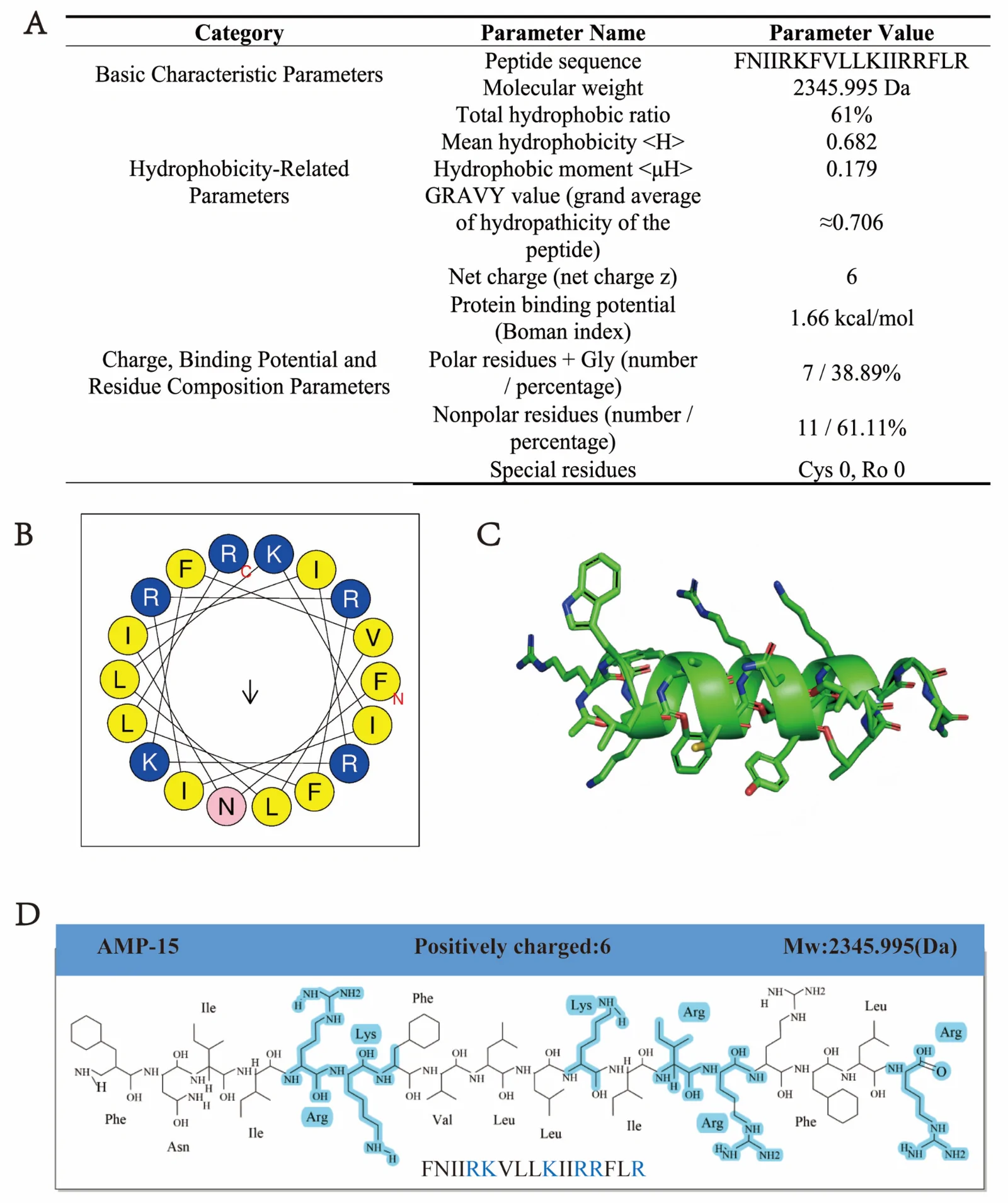
Physicochemical properties and structural characteristics of AMP-15. (**A**) Predicted physicochemical parameters of AMP-15, including amino acid sequence, molecular weight, hydrophobicity-related parameters, net charge, Boman index, and amino acid residue composition. (**B**) Helical wheel projection of AMP-15 showing the spatial distribution of hydrophobic residues and positively charged residues. (**C**) Predicted three-dimensional structure of AMP-15 showing its α-helical conformation. (**D**) Schematic representation of the amino acid sequence and chemical structure of AMP-15. The positively charged amino acid residues Lys and Arg are highlighted. Mw indicates molecular weight.

To preliminarily analyze the structural basis of AMP-15, its amino acid sequence, physicochemical properties, and spatial structural features were predicted. The results showed that the amino acid sequence of AMP-15 was FNIIRKFVLLKIIRRFLR, consisting of 18 amino acid residues, with a molecular weight of 2345.995 Da. The total hydrophobic ratio of this peptide was 61%, the mean hydrophobicity was 0.682, the hydrophobic moment was 0.179, and the GRAVY value was approximately 0.706. AMP-15 had a net charge of +6 and a Boman index of 1.66 kcal/mol. Among its residues, polar residues and glycine accounted for 7 residues, representing 38.89%, whereas non-polar residues accounted for 11 residues, representing 61.11%. No cysteine or special residues were present in the sequence (Figure 1A).

The helical wheel projection showed that the hydrophobic residues and positively charged residues of AMP-15 displayed a partially segregated spatial distribution, suggesting typical amphipathic structural characteristics (Figure 1B). Three-dimensional structure prediction showed that AMP-15 could form a predominantly α-helical conformation, with hydrophobic and charged residues distributed on different sides of the helix, which may facilitate its interaction with bacterial membranes (Figure 1C). Further sequence and structural schematic analysis showed that AMP-15 contained six positively charged amino acid residues, including Lys and Arg, which were mainly distributed in the middle and C-terminal regions of the peptide chain (Figure 1D). These results indicate that AMP-15 combines a high positive charge, strong hydrophobicity, and an amphipathic α-helical structural feature, which may provide a structural basis for its subsequent membrane-targeting antibacterial activity.

### 3.2. AMP-15 Exhibits Significant and Rapid Bactericidal Activity Against S. aureus

**Figure 2:**
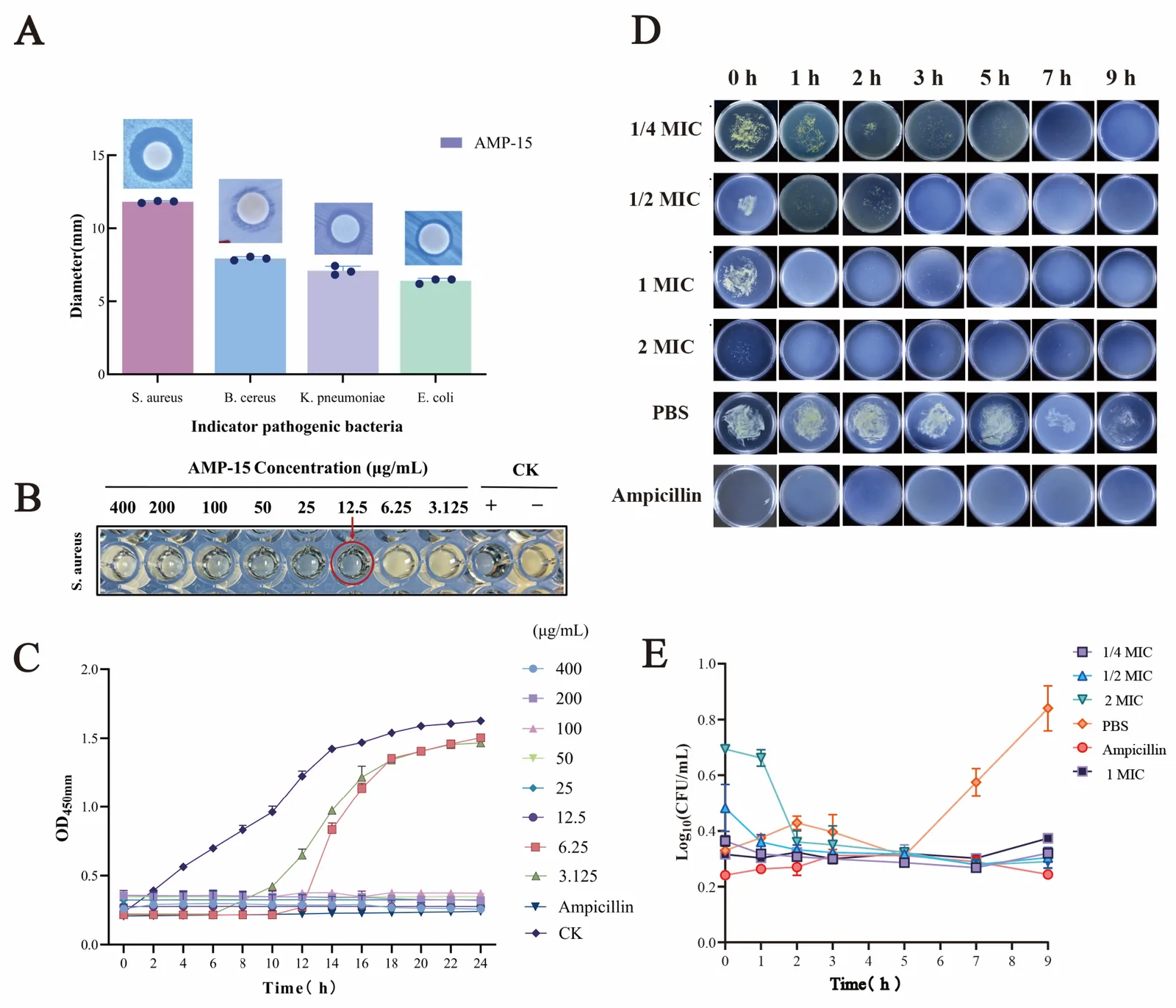
Antibacterial activity of AMP-15 and its inhibitory and bactericidal effects against *S. aureus.* (**A**) Diameter of the inhibition zones produced by AMP-15 against different indicator pathogens. (**B**) Minimum inhibitory concentration (MIC) of AMP-15 against *S. aureus* determined by the broth microdilution method. The MIC was defined as the lowest concentration of AMP-15 at which no obvious visible bacterial growth was observed and the OD_600_ value was close to that of the blank control. (**C**) Growth curves of *S. aureus* after treatment with different concentrations of AMP-15. Curves from top to bottom (as labelled in the legend): 400, 200, 100, 50, 25, 12.5, 6.25, and 3.125 μg/mL AMP-15, ampicillin positive control, and untreated control (CK). (**D**) Representative plate images from the time–kill assay of AMP-15 against *S. aureus* at 0.5×, 1×, 2×, and 3× MIC, with PBS and ampicillin used as the negative and positive controls, respectively. (**E**) Quantitative analysis of bacterial survival in different treatment groups in the time–kill assay. Data are presented as the mean ± SD of at least three independent experiments.

The inhibition zone assay showed that AMP-15 produced visible zones against all three indicator strains, with diameters of ~11 mm (*S. aureus*), ~8 mm (*B. cereus*), ~7 mm (*K. pneumoniae*) and ~6 mm (*E. coli)*. Therefore, *Staphylococcus aureus* was selected for subsequent experiments (Figure 2A). The results determined in Mueller–Hinton broth demonstrated that the inhibitory effect was concentration-dependent: as the concentration of AMP-15 increased from 3.125 μg/mL to 400 μg/mL, the turbidity of the bacterial suspension gradually decreased. At 12.5 μg/mL, the bacterial suspension remained clear, and this concentration was defined as the minimum inhibitory concentration (MIC) (Figure 2B). The assay performed in LB broth was used to compare the activity of the peptide under nutrient-rich conditions supporting optimal bacterial growth (Figures S1 and S2). The MIC value in LB broth was 50 μg/mL, and this concentration was adopted for subsequent experiments. Growth curve analysis showed that ≥12.5 μg/mL markedly inhibited proliferation; OD_600_ of 12.5–400 μg/mL remained low throughout, similar to ampicillin (Figure 2C). The time–kill assay confirmed rapid, concentration-dependent killing. Plate images showed that colonies decreased with increasing concentration and time. Viable counting showed that 1× MIC and 2× MIC reduced counts from the starting inoculum (~6.0 log_10_ CFU/mL) to ~2.0 log_10_ within 1–2 h, i.e., a ≥3 log_10_ reduction, meeting the criterion for bactericidal activity. The lower 0 h counts in the high-dose groups reflect rapid killing during the short interval between dosing and the first sampling (Figure 2D,E). These results demonstrate that AMP-15 reduces *S. aureus* viable counts in a concentration- and time-dependent manner.

### 3.3. AMP-15 Disrupts the Cell Membrane Integrity of S. aureus and Induces Intracellular ROS Accumulation

**Figure 3:**
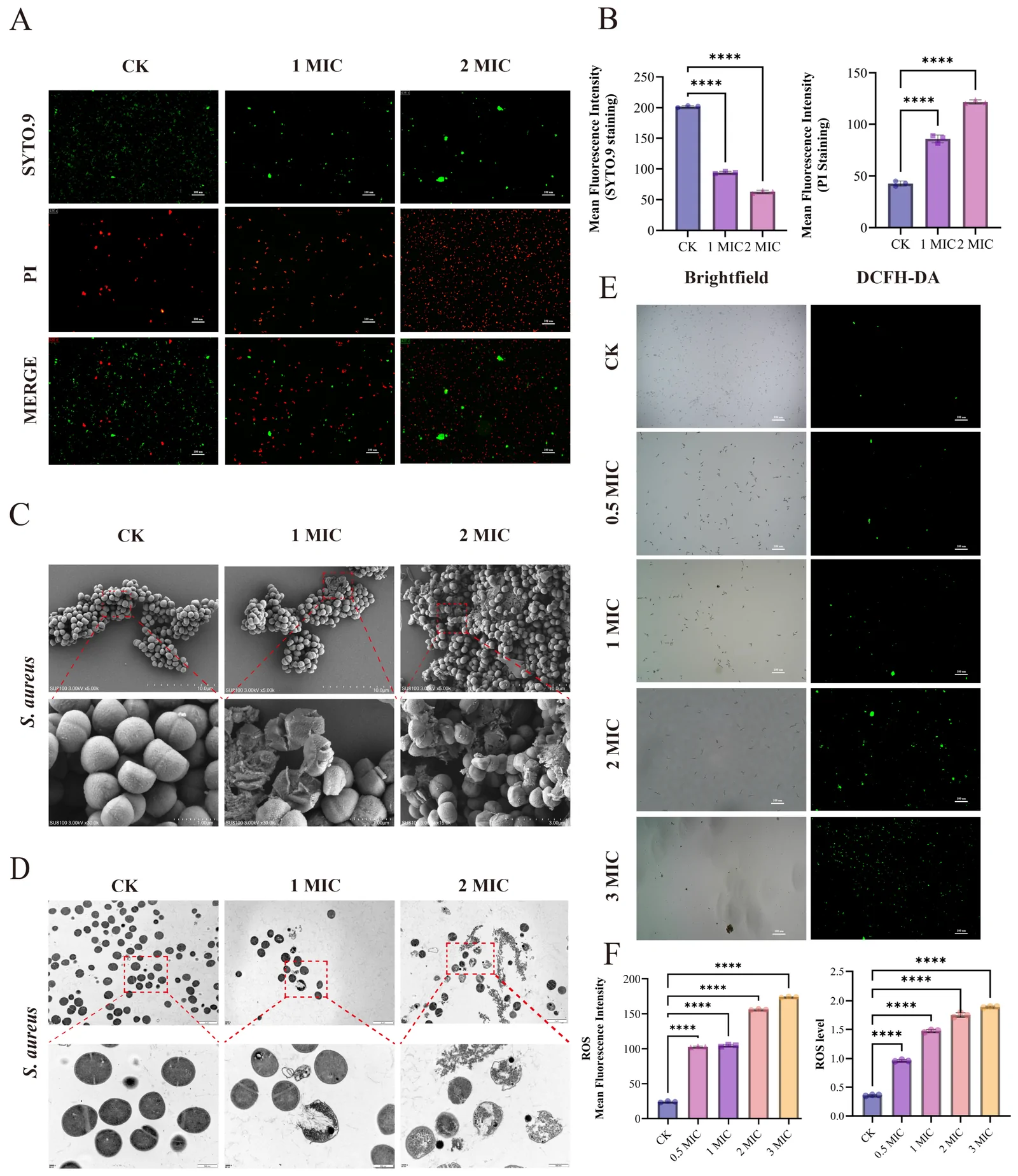
Effects of AMP-15 on cell membrane integrity, cell morphology, ultrastructure, and intracellular ROS accumulation in *S. aureus.* (**A**) SYTO 9/PI dual-staining fluorescence images of *S. aureus* after AMP-15 treatment. Green fluorescence indicates cells with intact membranes, whereas red fluorescence indicates cells with damaged membranes. (**B**) Quantitative analysis of the mean fluorescence intensities of SYTO 9 and PI. (**C**) Scanning electron microscopy images showing the surface morphology of *S. aureus* after AMP-15 treatment. (**D**) Transmission electron microscopy images showing the ultrastructural changes in *S. aureus* after AMP-15 treatment. (**E**) Fluorescence images of intracellular ROS detected by the DCFH-DA probe after treatment with different concentrations of AMP-15. (**F**) Quantitative analysis of the mean fluorescence intensity and relative intracellular ROS levels. CK indicates the untreated control group. Data are presented as the mean ± SD of at least three independent experiments. **** *p* < 0.0001. The red dashed line can be supplemented to indicate the partially enlarged area.

To investigate the antibacterial mechanism of AMP-15 against *S. aureus*, the effect of AMP-15 on cell membrane integrity was first evaluated using SYTO 9/PI dual staining. Fluorescence microscopy showed that the CK (0× MIC) group mainly exhibited strong green fluorescence with only a small amount of red fluorescence, indicating that most cells maintained intact membrane structures. After AMP-15 treatment, the green fluorescence decreased markedly, whereas the red fluorescence gradually increased, with the 1× MIC group showing the strongest red fluorescence signal (Figure 3A). Quantitative analysis of fluorescence intensity further demonstrated that AMP-15 treatment significantly decreased the mean fluorescence intensity of SYTO 9 while significantly increasing the mean fluorescence intensity of PI (*p* < 0.0001) (Figure 3B). Since PI can only enter membrane-damaged cells and emit red fluorescence upon binding to nucleic acids, these results indicate that AMP-15 markedly disrupts the cell membrane integrity of *S. aureus* and increases membrane permeability.

Scanning electron microscopy (SEM) was further used to observe the effect of AMP-15 on bacterial surface morphology. In the CK group, *S. aureus* cells displayed a typical spherical shape, with smooth surfaces, intact structures, and relatively regular grape-like aggregation. After treatment with 1/2× MIC AMP-15, some cells showed surface shrinkage, deformation, and collapse. After treatment with 1× MIC, the structural damage became more pronounced, and the cell surfaces appeared rough and irregular, with some cells showing rupture or fragmentation tendencies (Figure 3C). Transmission electron microscopy (TEM) observations were consistent with the SEM results. Cells in the CK group had clear cell wall and membrane boundaries and uniformly distributed intracellular electron density. At 1/2× MIC, the ultrastructure was subtly altered. Pronounced ultrastructural alterations were observed at 1× MIC, including blurred and disrupted cell wall and membrane structures, cytoplasmic leakage, intracellular vacuolization, and uneven distribution of intracellular contents (Figure 3D). These results indicate that, at the ultrastructural level, AMP-15 disrupts the cell envelope of *S. aureus* mainly at the higher (1× MIC) concentration.

To further determine whether AMP-15 induces intracellular oxidative stress, ROS levels were detected using the DCFH-DA probe. Fluorescence microscopy showed that only weak green fluorescence was observed in the CK group, whereas AMP-15 treatment gradually enhanced green fluorescence, and the signal became more evident with increasing treatment concentration. The strongest fluorescence signals were observed in the 1× MIC and 2× MIC groups (Figure 3E). Quantitative analysis showed that, compared with the CK group, treatment with 0.5× MIC, 1/2× MIC, 1× MIC, and 2× MIC AMP-15 significantly increased ROS fluorescence intensity and relative ROS levels (*p* < 0.0001), showing an overall concentration-dependent upward trend (Figure 3F). Taken together, these results indicate that the antibacterial activity of AMP-15 against *S. aureus* is closely associated with disruption of cell membrane integrity, morphological and ultrastructural damage, and intracellular ROS accumulation.

### 3.4. Molecular Docking Analysis Between AMP-15 and S. aureus Thymidylate Kinase (TMK)

**Figure 4:**
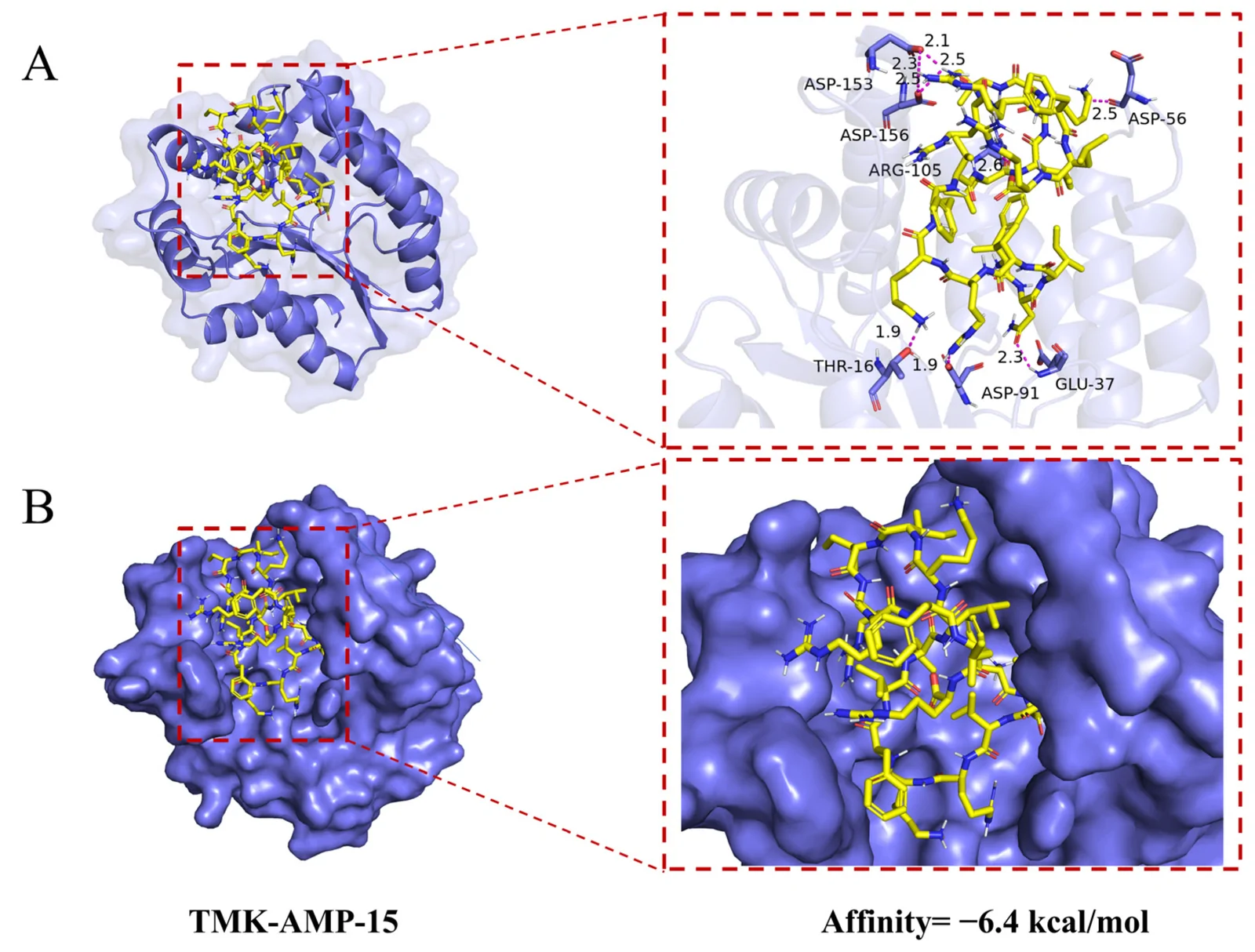
Molecular docking analysis of AMP-15 with *S. aureus* thymidylate kinase (TMK). (**A**) Overall binding mode of AMP-15 with TMK and an enlarged view of the local interactions. TMK is shown in blue, and AMP-15 is shown in yellow. The enlarged view illustrates the hydrogen bonds and other non-covalent interactions between AMP-15 and key amino acid residues within the TMK binding pocket, together with the corresponding interaction distances. (**B**) Surface representation of the binding mode of AMP-15 with TMK and an enlarged local view. AMP-15 is embedded within the concave binding region on the TMK surface and exhibits a certain degree of spatial complementarity with the binding pocket. Molecular docking analysis predicted a binding affinity of −6.4 kcal/mol for the TMK–AMP-15 complex.

To further explore whether AMP-15 may have a potential intracellular target, molecular docking was performed to analyze the interaction between AMP-15 and *S. aureus* thymidylate kinase (TMK). The docking results showed that AMP-15 could insert into the binding pocket of *S. aureus* TMK and form hydrogen bonds or other non-covalent interactions with residues such as ASP-153, ASP-156, ARG-105, THR-16, ASP-91, GLU-37, and ASP-56, with interaction distances mainly concentrated at approximately 1.9–2.5 Å (Figure 4A). The surface model further showed that the binding region between AMP-15 and TMK displayed a certain degree of spatial complementarity, and the predicted binding affinity of the TMK–AMP-15 complex was −6.4 kcal/mol. These results suggest a possible binding interaction between AMP-15 and TMK; however, this interaction still requires further validation through in vitro binding assays or enzyme activity inhibition assays.

### 3.5. AMP-15 Inhibits S. aureus Biofilm Formation and Attenuates Mature Biofilms

**Figure 5:**
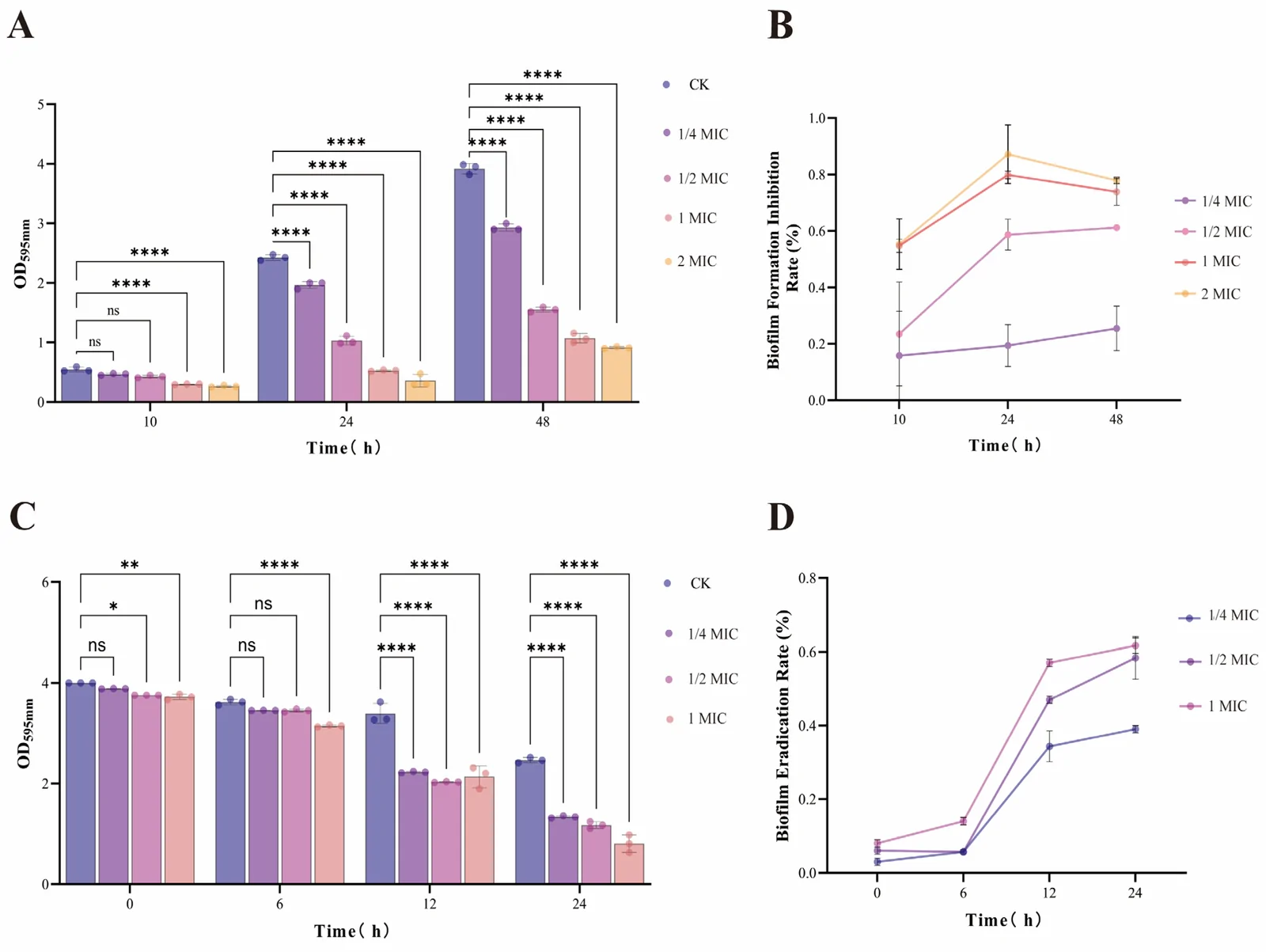
Effects of AMP-15 on biofilm formation and mature biofilms of *S. aureus*. (**A**) Biofilm formation of *S. aureus* after treatment with different concentrations of AMP-15 for 10, 24, and 48 h, as determined by crystal violet staining. (**B**) Inhibition rates of AMP-15 against biofilm formation at different time points. (**C**) Residual biofilm biomass of mature biofilms after treatment with AMP-15 for 0, 6, 12, and 24 h, as determined by crystal violet staining. (**D**) Eradication rates of AMP-15 against mature biofilms. Data are presented as the mean ± SD of at least three independent experiments. ns indicates no significant difference; * *p* < 0.05, ** *p* < 0.01, **** *p* < 0.0001.

To evaluate the effect of AMP-15 on *Staphylococcus aureus* biofilms, this study investigated its impacts on biofilm formation and preformed biofilms. The crystal violet quantification results showed that the biofilm biomass in the control group (CK group) kept increasing with prolonged incubation time, and the absorbance at 595 nm (OD_595_) increased significantly at 24 h and 48 h. Compared with the control group, AMP-15 treatment reduced biofilm biomass in a concentration-dependent manner (Figure 5A). At 24 h, the 1× minimum inhibitory concentration (1× MIC) group and the 2× minimum inhibitory concentration (2× MIC) group exhibited the strongest inhibitory effects, with inhibition rates of approximately 80% (Figure 5B). These two groups still maintained inhibitory effects at 48 h. Since 1× MIC are bactericidal concentrations, the biofilms almost disappeared at these concentrations, which was mainly attributed to the killing of viable bacteria rather than a specific anti-biofilm mechanism.

This study further adopted the crystal violet quantification assay (measuring OD_595_) to assess the effect of AMP-15 on preformed (mature) biofilms. The residual biofilm biomass gradually decreased as the treatment duration increased: the weakening effect emerged after 6 h of treatment, and all concentration groups reduced the OD_595_ values at 12 h and 24 h (Figure 5C). The variation trend of the eradication rate was consistent with OD_595_, and the overall biofilm eradication effect was time-dependent; the eradication rate reached approximately 40–60% at 24 h (Figure 5D). As crystal violet detects the total biofilm biomass (bacterial cells plus extracellular matrix), this result reflects a reduction in total biomass rather than direct quantification of dead bacterial cells.

### 3.6. AMP-15 Shows Moderate Biocompatibility and Physicochemical Stability

**Figure 6:**
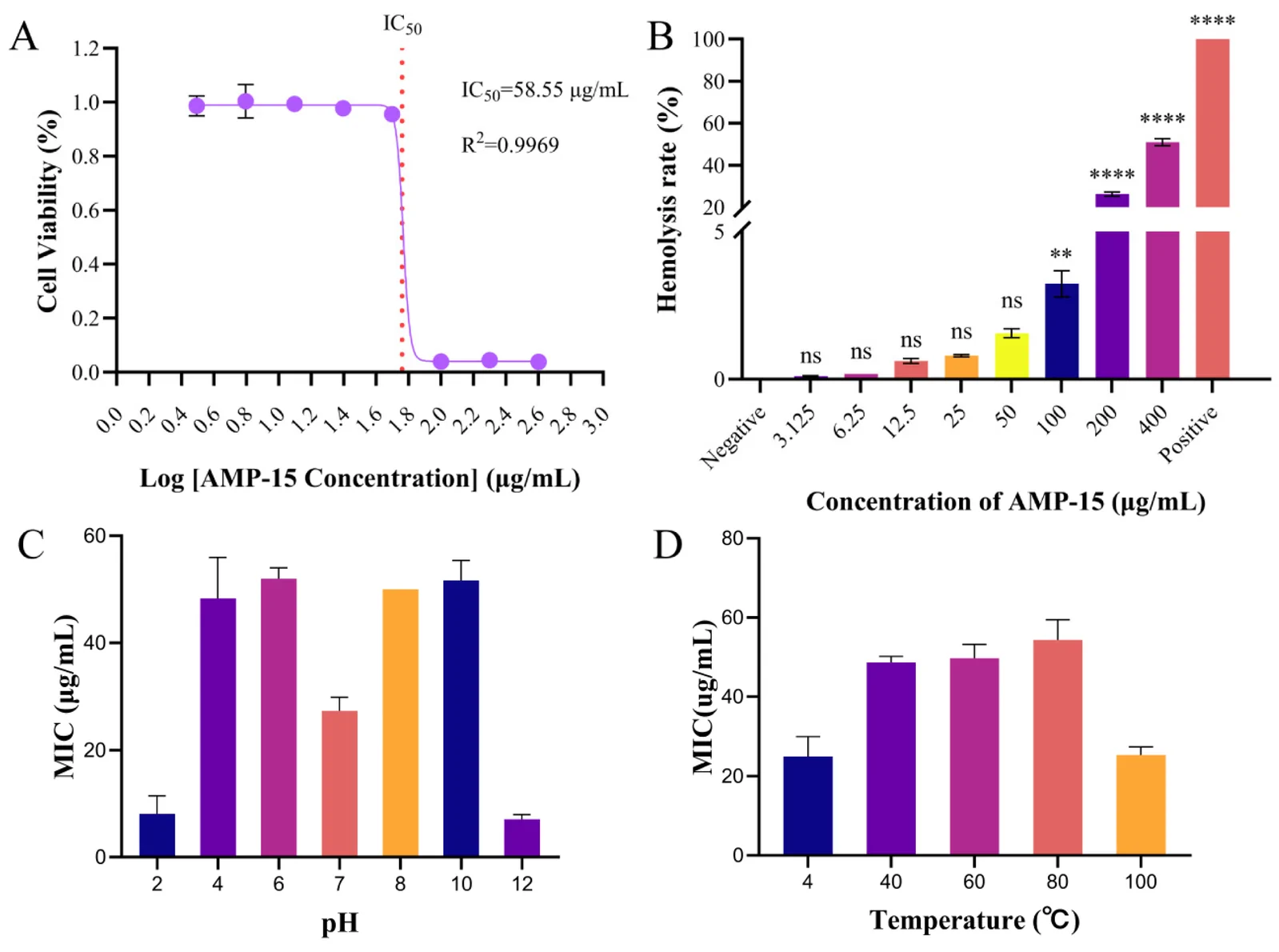
Biocompatibility and physicochemical stability of AMP-15. (**A**) Effect of AMP-15 on the viability of 293T cells. Cell viability was measured after treatment with different concentrations of AMP-15, and the calculated IC_50_ was 58.55 μg/mL, with a goodness of fit R^2^ of 0.9969. (**B**) Hemolytic activity of AMP-15 at different concentrations. The negative and positive controls represent 0% and 100% hemolysis, respectively. (**C**) Antibacterial activity of AMP-15 after treatment under different pH conditions, expressed as MIC values. (**D**) Antibacterial activity of AMP-15 after treatment at different temperatures, expressed as MIC values. Data are presented as the mean ± SD of at least three independent experiments. ns indicates no significant difference; ** *p* < 0.01, **** *p* < 0.0001.

To evaluate the biocompatibility of AMP-15 peptide with mammalian cells, its effect on the viability of 293T cells was first examined. The results showed that, at relatively low concentrations, AMP-15 had only a limited effect on 293T cell viability, and cell viability remained at a relatively high level. However, as the concentration of AMP-15 further increased, cell viability decreased rapidly, indicating clear concentration-dependent cytotoxicity. Dose–response curve fitting showed that the IC_50_ of AMP-15 against 293T cells was 58.55 μg/mL, with a goodness of fit R^2^ of 0.9969 (Figure 6A).

The hemolysis assay showed that AMP-15 had a low hemolysis rate in the concentration range of 3.125–50 μg/mL, with no significant difference or only a slight increase compared with the negative control. When the concentration increased to 100 μg/mL, the hemolysis rate increased significantly. At 200 and 400 μg/mL, the hemolysis rate further increased, with the 400 μg/mL group reaching approximately 50%. The hemolysis rate of the positive control group was close to 100% (Figure 6B). These results indicate that AMP-15 exhibits relatively good hemocompatibility at low to moderate concentrations, although a certain hemolytic risk remains at high concentrations.

The antibacterial stability of AMP-15 under different pH and temperature conditions was further investigated. The pH stability assay showed that, after treatment at pH 2.0/12.0, the MIC was 6.25 μg/mL, indicating that it retained strong antibacterial activity under strongly acidic and strongly alkaline conditions. After treatment at pH 7, the MIC was approximately 25 μg/mL, whereas the MIC increased to approximately 50 μg/mL after treatment at pH 6, 8, and 10, suggesting that its antibacterial activity decreased after exposure to neutral to partially weakly acidic or weakly alkaline conditions (Figure 6C). The thermal stability assay showed that the MIC of AMP-15 was approximately 25 μg/mL after treatment at 4 °C and 100 °C, whereas it increased to approximately 50 μg/mL after treatment at 40, 60, and 80 °C (Figure 6D). These results indicate that AMP-15 has a certain degree of pH and thermal stability, although its antibacterial activity is still affected by treatment conditions.

### 3.7. AMP-15 Significantly Reduces S. aureus Counts in Chicken and Milk Matrix Models

**Figure 7:**
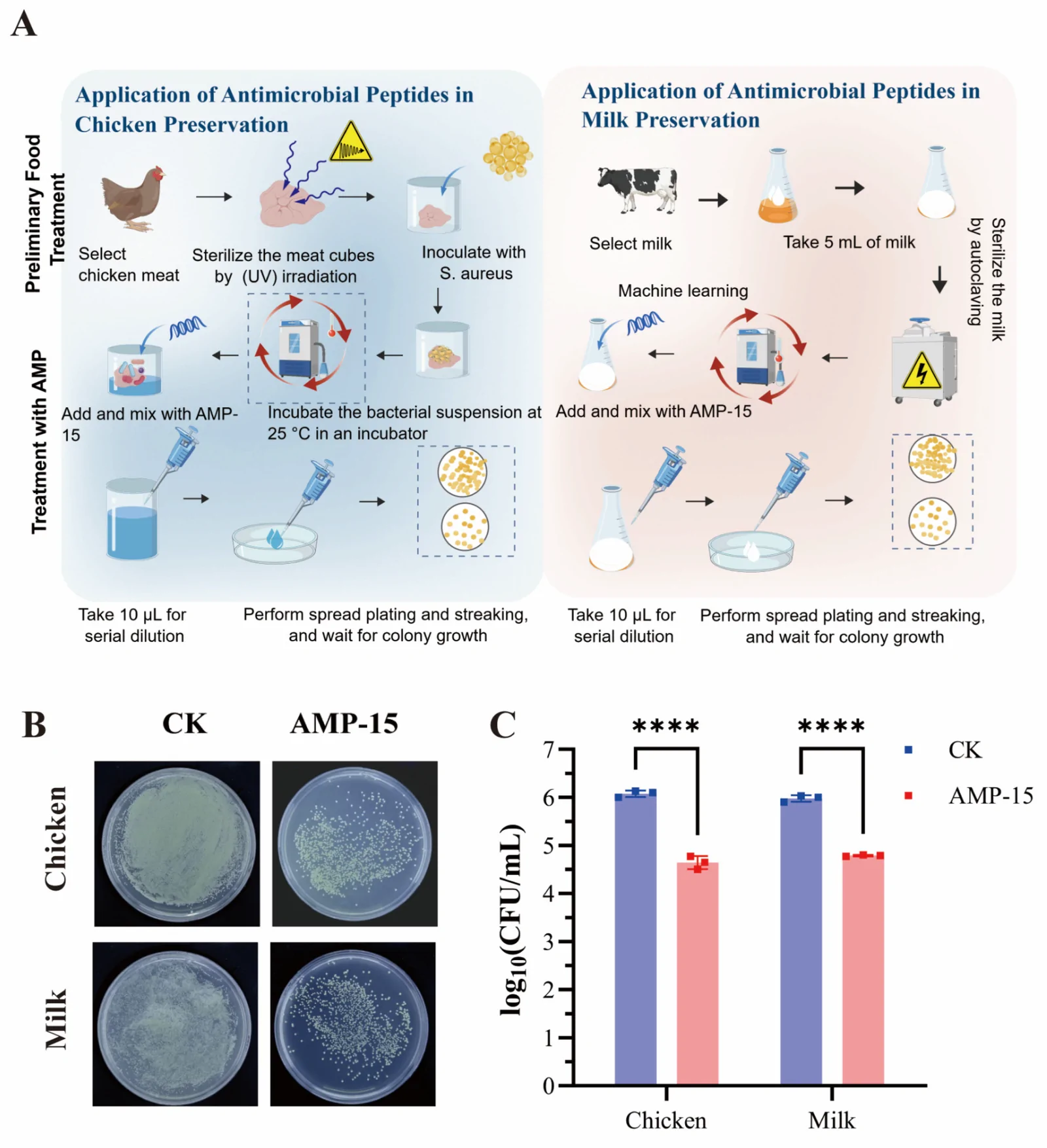
Inhibitory effect of AMP-15 against *S. aureus* in chicken and sterilized milk contamination models. (**A**) Schematic illustration of the experimental workflow for evaluating the antibacterial activity of AMP-15 against *S. aureus* in chicken meat and sterilized milk contamination models. (**B**) Representative plate images of *S. aureus* recovered from chicken and sterilized milk samples in the CK and AMP-15-treated groups. (**C**) Changes in viable *S. aureus* counts in chicken and sterilized milk samples after AMP-15 treatment. Results for chicken samples are expressed as log_10_(CFU/g), while results for sterilized milk samples are expressed as log_10_(CFU/mL). CK indicates the untreated control group. Data are presented as the mean ± SD of at least three independent experiments, **** *p* < 0.0001. Figure 7A was created with BioGDP (https://www.biogdp.com, accessed on 6 May 2026) [24].

To further evaluate the antibacterial efficacy of AMP-15 in food matrices, *S. aureus* contamination models were established using chicken and sterilized milk. Representative plate images showed abundant colony growth in both chicken and sterilized milk samples from the CK group, whereas the number of colonies was markedly reduced after AMP-15 treatment in both food matrices. These results indicate that AMP-15 retained antibacterial activity in animal-derived food matrices (Figure 7A).

Quantitative analysis showed that, in the chicken model, the *S. aureus* count in the CK group was approximately 6.1 log_10_(CFU/g), whereas AMP-15 treatment reduced it to approximately 4.6–4.7 log_10_(CFU/g). In the sterilized milk model, the bacterial count in the CK group was approximately 6.0 log_10_(CFU/mL), and AMP-15 treatment reduced it to approximately 4.8 log_10_(CFU/mL) (Figure 7B,C). Compared with the CK group, AMP-15 treatment significantly reduced the number of *S. aureus* cells in both the chicken and sterilized milk contamination models (*p* < 0.0001). These results demonstrate that AMP-15 maintained pronounced anti-*S. aureus* activity in chicken and dairy matrices.

## 4. Discussion

In this study, AMP-15, an 18-residue cationic peptide (net charge +6) with high hydrophobicity (0.682) and an amphipathic α-helical conformation exhibited rapid bactericidal activity against *S. aureus* (MIC = 12.5 μg/mL) and reduced viable counts to approximately 2.0–2.5 log_10_ CFU/mL within 2 h at 1×–2× MIC. Its activity was associated with cell membrane disruption, intracellular ROS accumulation, and a potential interaction with thymidylate kinase (TMK); it also inhibited biofilm formation (up to ~80% at 2× MIC) and reduced mature biofilm biomass (40–60% eradication at 24 h), while showing moderate biocompatibility (IC_50_ = 58.55 μg/mL; low hemolysis at ≤50 μg/mL) and condition-dependent stability. These findings are discussed below in comparison with related studies.

The hydrophobicity of AMP-15 (0.682) was higher than that of LL-37, nisin, and melittin, as calculated using the same parameters. Hydrophobicity and amphipathic α-helical structure are key determinants of membrane binding, insertion, and permeabilization by AMPs [25,26]. Chen et al. [26] showed that increasing the hydrophobicity of α-helical AMPs enhanced membrane permeabilization and antimicrobial potency, but also increased hemolytic activity. Edwards et al. [27] similarly reported that β-hairpin peptides with higher hydrophobicity and amphipathicity showed stronger antimicrobial activity but greater cytotoxicity. In the present study, the relatively high hydrophobicity of AMP-15 likely contributes to its strong membrane interaction, consistent with its low MIC (12.5 μg/mL); however, this property may also explain the sharp cytotoxicity observed at ≥100 μg/mL (293T viability ~5%), whereas hemolysis remained low at ≤50 μg/mL. These results indicate that the activity–safety balance of AMP-15 must be carefully evaluated.

Zhang et al. [28] reported that the chickpea-derived modified peptides KTA and KTR maintained cationic and amphipathic properties, exhibited potent inhibitory activity against *S. aureus*, and inactivated bacteria by disrupting the cell membrane and interfering with peptidoglycan synthesis. The MIC of AMP-15 (12.5 μg/mL) was comparable to that of KTA and KTR, and its mechanism similarly involves membrane disruption. In addition, the stronger activity of AMP-15 against *S. aureus* (inhibition zone ~11 mm) than against *K. pneumoniae* (~7 mm) may be partly explained by the abundance of negatively charged lipoteichoic acids in the *S. aureus* cell wall, which provide a strong initial adsorption interface for cationic peptides.

Mechanistic analyses using PI/SYTO 9 staining, SEM, and TEM showed that AMP-15 significantly increased membrane permeability and disrupted cell envelope integrity, with the 1× MIC group showing the strongest PI signal (*p* < 0.0001). Sun et al. [29] reported that the moringa seed-derived peptide MOp3 caused irreversible membrane damage in *S. aureus*, accompanied by ROS accumulation, DNA fragmentation, and metabolic disturbance. Zhang et al. [30] showed that the amphipathic peptide A24 increased membrane permeability and elevated intracellular ROS levels in *S. aureus*. In the present study, AMP-15 induced a concentration-dependent increase in ROS (*p* < 0.0001 at 1/4×–2× MIC), and membrane disruption was evident at 1/2×–1× MIC; these observations are consistent with those reported for MOp3 and A24. This suggests that the bactericidal process of AMP-15, like that of MOp3 and A24, involves both direct membrane damage and oxidative stress. However, because ROS accumulation may also be a secondary consequence of membrane damage and metabolic imbalance, it is more appropriately interpreted as a contributing factor rather than the sole or primary cause of bacterial death.

It should be noted that the DCFH-DA probe applied in this work only reflects the overall relative changes in intracellular oxidative stress and cannot distinguish specific ROS subtypes. Future work will adopt more specific fluorescent probes, including Amplex Red coupled with horseradish peroxidase for hydrogen peroxide detection, dihydroethidium for superoxide anion measurement, and aminophenylfluorescein for hydroxyl radical quantification, to precisely identify which ROS are elevated upon AMP-15 treatment.

Molecular docking predicted that AMP-15 binds to *S. aureus* TMK with a binding affinity of −6.4 kcal/mol, forming hydrogen bonds or other non-covalent interactions with ASP-153, ASP-156, ARG-105, THR-16, ASP-91, GLU-37, and ASP-56 at distances of 1.9–2.5 Å. Martínez-Botella et al. [31] developed sulfonylpiperidine inhibitors of Gram-positive TMK that showed relatively high selectivity over the human TMK homolog. Hu et al. [32] constructed the TMK-targeting hybrid peptide SWP, which specifically bound bacterial TMK and reduced bacterial burden in an in vivo infection model. Compared with these TMK-targeting molecules, the predicted binding affinity of AMP-15 (−6.4 kcal/mol) suggests a potential binding interaction; however, this remains a computational prediction and requires validation by enzyme activity inhibition assays, isothermal titration calorimetry, or other binding assays.

The present study showed that AMP-15 inhibited *S. aureus* biofilm formation by ~80% and ~90% at 1× and 2× MIC (24 h), respectively, and achieved eradication rates of 40–60% against mature biofilms at 24 h, while also reducing viable cells within mature biofilms at 1×–2× MIC. Akhash et al. [33] designed the sapecin B-derived peptide mKLK, which exhibited stronger inhibitory, eradication, and dispersal effects against MRSA and MSSA biofilms than the parent peptide, including potent antibiofilm activity at sub-MICs. Jin et al. [34] reported that FAM286 at non-bactericidal concentrations inhibited biofilm formation and eradicated mature biofilms, potentially through interactions with biofilm-associated proteins such as SarA, AgrA, and Hld. The antibiofilm activity of AMP-15 aligns with these reports, supporting the potential of cationic amphipathic peptides to target both planktonic and biofilm-embedded *S. aureus*. From a food-processing perspective, this antibiofilm activity is more application-relevant than MIC alone, because mature biofilms on equipment and food-contact surfaces are difficult to remove in practice.

Safety and stability are key factors for food-preservation candidates. In this study, AMP-15 showed low hemolysis at 3.125–50 μg/mL, but significant hemolysis at ≥100 μg/mL (approximately 50% at 400 μg/mL), and its IC_50_ against 293T cells was 58.55 μg/mL (R^2^ = 0.9969). Gou et al. [24] reported that an anoplin-derived AMP exhibited concentration-dependent cytotoxicity and hemolytic activity at relatively high concentrations. Gagat et al. [22] reviewed that increasing charge and hydrophobicity generally enhances antimicrobial activity but also increases cytotoxicity, which is consistent with our observation that the +6 charge and high hydrophobicity of AMP-15 contribute to both its antimicrobial efficacy and its cytotoxicity at high concentrations. Jiao et al. [21] and León Madrazo et al. [23] noted that industrial AMP applications face challenges [35] in biosafety, food-matrix compatibility, and large-scale production, and that application potential cannot be judged solely by in vitro MIC values. Our results support this view: although AMP-15 retained strong activity after exposure to pH 2 and pH 12 (MIC ~ 6.25 μg/mL) and at 4 °C and 100 °C (MIC ~ 25 μg/mL), its MIC increased to ~50 μg/mL after treatment at pH 6, 8, and 10 or at 40, 60, and 80 °C, indicating that its stability is condition-dependent. Therefore, the selectivity index and effective/safe dose range of AMP-15 require further clarification.

In real food matrices, AMP efficacy can be reduced by proteins, lipids, salts, and enzymes [24]. Sun et al. [29] demonstrated that MOp3 disrupted *S. aureus* membranes and inhibited its growth in pasteurized milk, underscoring the need for food matrix validation. In the present study, AMP-15 at 1× MIC (50 μg/mL) reduced *S. aureus* counts from 6.1 to 4.6–4.7 log_10_ CFU/g in chicken (a ~1.4–1.5 log_10_ reduction) and from 6.0 to 4.8 log_10_ CFU/mL in sterilized milk (a ~1.2 log_10_ reduction), indicating that AMP-15 retains antibacterial activity in animal-derived food matrices. However, the milk model used sterilized milk, and the assays were short-term; thus, the results do not fully simulate real storage conditions (natural microbiota, low temperature, enzymatic changes, and quality deterioration). Further validation under more realistic conditions is therefore needed.

Overall, AMP-15 combines strong anti-*S. aureus* activity (MIC 50 μg/mL), membrane disruption- and oxidative stress-associated bactericidal mechanisms, antibiofilm capacity (up to ~90% inhibition and 45–68% eradication), moderate biocompatibility (IC_50_ 58.55 μg/mL; low hemolysis at ≤50 μg/mL), and antibacterial efficacy in chicken and milk matrices. These properties make it a potential candidate molecule for controlling *S. aureus* contamination in foods.

## 5. Conclusions

This study systematically evaluated the antibacterial activity and mechanism of action of the antimicrobial peptide AMP-15 against *S. aureus*. The results showed that AMP-15 exhibited strong inhibitory and bactericidal activity against *S. aureus*. Its antibacterial action was associated with disruption of cell membrane integrity, intracellular ROS accumulation, and a potential interaction with thymidylate kinase (TMK), suggesting a mechanism involving membrane damage and possible intracellular effects. In addition, AMP-15 inhibited biofilm formation and reduced the number of viable cells within mature biofilms. It also showed significant antibacterial efficacy in chicken and milk contamination models. Preliminary safety evaluation indicated that AMP-15 had a certain degree of biocompatibility at low to moderate concentrations. Overall, these results suggest that AMP-15 has potential as a novel peptide-based candidate for food preservation and provide experimental evidence for the further development of antimicrobial peptides in food preservation applications.

## 6. Patents

A patent application related to AMP-15 (Application No.: 2026106398227, filing date: 11 May 2026) has been filed by Xinjiang Academy of Agricultural Sciences. This patent application is currently accepted by CNIPA and has not yet been published.

## Data Availability

The original contributions presented in this study are included in the article/Supplementary Materials. Further inquiries can be directed to the corresponding authors.

## Supplementary Materials

The following supporting information can be downloaded at https://www.mdpi.com/article/10.3390/foods15213990/s1. Figure S1: MIC 96-well plate diagram of AMP-15 against *Staphylococcus aureus* in LB broth. The MIC was defined as the lowest concentration of AMP-15 at which no obvious visible bacterial growth was observed; Figure S2: Growth curves of *S. aureus* after treatment with different concentrations of AMP-15. OD_450_ was monitored over 24 h to evaluate the inhibitory effect of AMP-15 on the growth of *S. aureus*.

## Abbreviations

The following abbreviations are used in this manuscript:

AMP	Antimicrobial peptide
MIC	Minimum inhibitory concentration
MBC	Minimum bactericidal concentration
ROS	Reactive oxygen species
DCFH-DA	2′,7′-dichlorodihydrofluorescein diacetate
TMK	Thymidylate kinase
LB	Luria–Bertani
PBS	Phosphate-buffered saline
OD	Optical density
SEM	Scanning electron microscopy
TEM	Transmission electron microscopy
GRAVY	Grand average of hydropathicity
IC_50_	Half maximal inhibitory concentration
Aa	Amino acid
RCSB PDB	Research Collaboratory for Structural Bioinformatics Protein Data Bank
CCK-8	Cell Counting Kit-8
MSSA	Methicillin-resistant
