# Supplementary material for: Antibacterial and Antibiofilm Activity of AMP-15 Against Staphylococcus aureus and Its Potential Application in Food Matrices
Source: Foods. 2026 Nov 6;15(21):3990. doi: 10.3390/foods15213990 (PMC13763395; doi:10.3390/foods15213990)
Supplement: Supplementary file 1 [file foods-15-03990-s001.zip › foods-4610438-supplementary.pdf]

Supplementary Materials

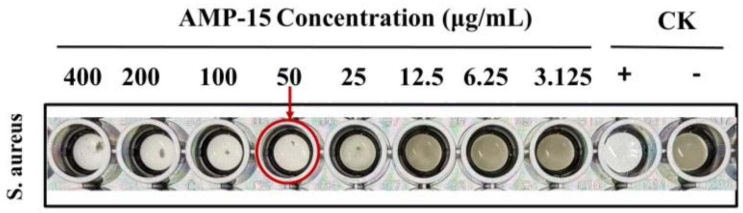

Figure S1. MIC 96-well plate diagram of AMP-15 against *Staphylococcus aureus* in LB (Luria-Bertani) broth. The MIC was defined as the lowest concentration of AMP-15 at which no obvious visible bacterial growth was observed.

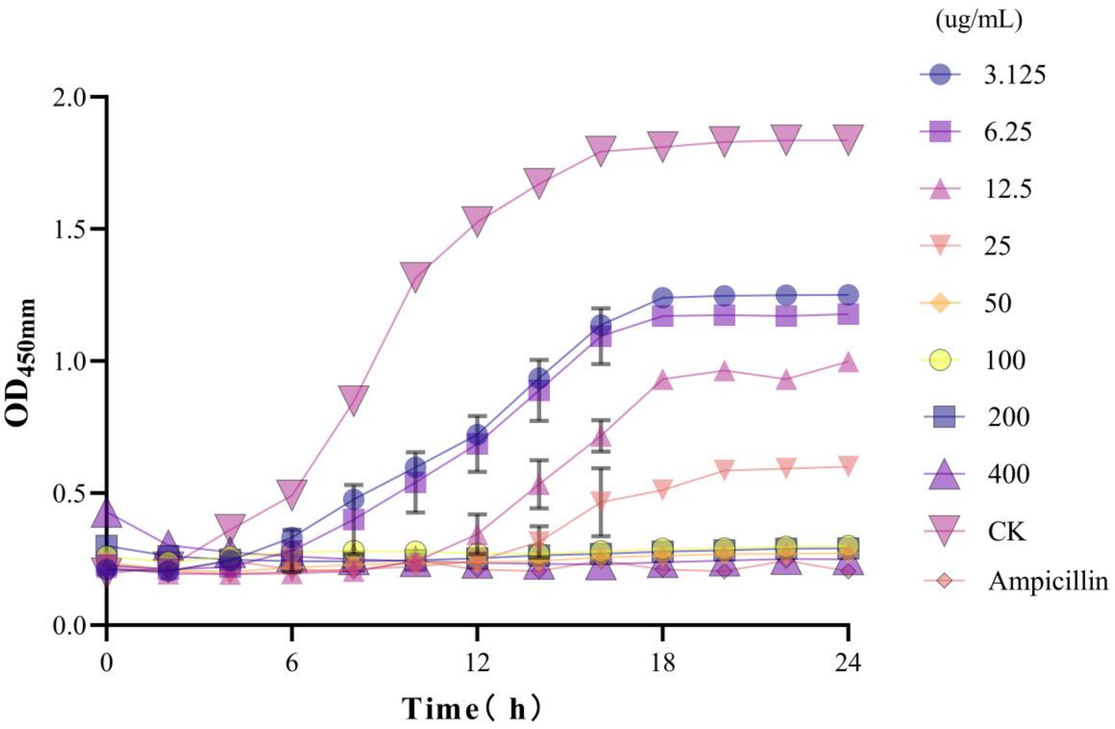

Figure S2. Growth curves of *S. aureus* after treatment with different concentrations of AMP-15. OD_{450} was monitored over 24 h to evaluate the inhibitory effect of AMP-15 on the growth of *S. aureus*.
